# The configurational pathways associated with executive function in preschool children: An fsQCA analysis based on gross motor skill development

**DOI:** 10.1371/journal.pone.0361476

**Published:** 2026-10-26

**Authors:** Dongbin Lai, Zhipeng Zhao, Zhi Li, Jinsong Wu, Pengfei Wen

**Affiliations:** School of Physical Education, Guangzhou Sport University, Guangzhou, China; Damghan University, IRAN, ISLAMIC REPUBLIC OF

## Abstract

The preschool years are a critical period for the rapid development of executive function in children. While executive function in preschool children is influenced by multiple factors, existing research has been limited by its theoretical and empirical perspectives, resulting in singular pathways for promoting executive function. To address this, the study adopted a systems theory perspective to explore the configurational pathways through which subsystems of gross motor skills are associated with higher levels of executive function in preschoolers. The study included test data from 67 children aged 3–6 years. Using fuzzy-set qualitative comparative analysis (fsQCA), six subsystems of gross motor skills (running, jumping, throwing, catching, kicking, and striking) were treated as antecedent conditions to explore the configurational associations of gross motor skill subsystems with executive function. Necessary condition analysis indicated that no single gross motor skill subsystem constituted a necessary condition for higher executive function in preschool children. Subsequent configurational analysis identified three distinct pathways that are associated with higher levels of executive function (with a total consistency of 0.906 and an overall coverage of 0.496). The development of executive function in preschool children is not associated with single gross motor actions in a linear manner but rather with multiple concurrent combinations of gross motor skill subsystems characterized by “multiple concurrent pathways leading to the same goal.” This is reflected in the three configurational pathways: “a combination of jumping, throwing, and striking actions,” “a combination of running, kicking, and striking actions,” and “a combination of running, throwing, and catching actions.” These findings provide preliminary insights and hypothesis-generating evidence for future research on gross motor skill activities and executive function development in preschool children.

## 1. Introduction

Executive Function (EF) is a higher-order cognitive process involved in the regulation of goal-directed behaviors. It primarily includes inhibitory control, working memory, and cognitive flexibility [1]. Higher levels of EF are associated with children’s academic performance, mental health, and social behaviors. Research indicates that children with well-developed EF exhibit higher levels of focus and task management abilities, and are associated with better academic outcomes [2,3]. Additionally, EF is closely related to children’s emotional regulation skills, with stronger EF being associated with better emotional regulation abilities and lower levels of anxiety and depression symptoms [4]. In terms of social behaviors, children with strong EF demonstrate greater social adaptability and prosocial behaviors, which are associated with greater understanding of social situations, fewer interpersonal conflicts, and more cooperative relationships [5]. Children aged 3–6 years represent a sensitive period for the development of executive function, during which inhibitory control, working memory, and cognitive flexibility all show rapid growth [6]. However, children with underdeveloped EF often exhibit impulsive behaviors, attention deficits, and maladaptive tendencies [7]. Several studies have also found that preschool children generally exhibit relatively low performance on EF tasks [8,9]. Therefore, investigating factors associated with EF performance in children aged 3–6 years is of important theoretical and practical significance.

Gross motor skills (GMS) refer to movements produced by the body’s large muscles or muscle groups, including fundamental movement skills such as running, jumping, throwing, catching, kicking, and striking. Research has shown that higher levels of GMS in early childhood are associated with future motor skills but is also closely associated with children’s cognitive development and social behavior [10]. Notably, ages 3–6 also represent a sensitive period for the development of GMS, during which fundamental movement skills rapidly emerge and become refined [11]. This dual sensitivity in development makes it particularly advantageous to examine the relationship between GMS and EF during this period.

In recent years, a growing body of cross-sectional studies has consistently reported a positive association between GMS and EF [12–14]. Intervention studies have further indicated that when physical activities incorporate cognitively demanding and complex movements may be associated with improvements in EF performance [15,16]. These complex movements are largely based on fundamental GMS, including jumping, running, kicking, and catching [17]. In addition, studies by Kesha (2022), Townsend (2007), and others have implemented interventions using multiple GMS as well as single motor skills (e.g., kicking), and found that higher levels of EF were observed following these interventions [18,19]. Furthermore, developmental research suggests that GMS and EF show overlapping developmental trajectories during early childhood, highlighting the importance of examining their relationship during this period [20]. Taken together, these findings suggest that ages 3–6 represent an optimal developmental period for examining the relationship between GMS and EF, and that their interaction during this period warrants further investigation.

Although previous studies have reported a positive association between GMS and EF, and some intervention studies have shown favorable outcomes using combinations of movements, several limitations remain. First, in terms of research content, existing studies have typically treated multiple movements as a unified intervention package and have often used overall GMS scores as the unit of analysis. While this approach can demonstrate overall associations or intervention outcomes, it makes it difficult to address more fine-grained questions, such as which specific movement combinations are more strongly associated with EF outcomes, whether combinations such as jumping, throwing, and striking differ from combinations such as running, kicking, and catching, and whether multiple equivalent pathways exist. Second, in terms of analytical paradigms, Existing research has primarily adopted a variable-centered analytical framework, focusing mainly on the independent associations between individual motor skills or overall motor competence and executive function, while paying less attention to the complex combination patterns formed by multiple motor skill domains from a configurational perspective. Although methods such as multiple regression with interaction terms, structural equation modeling, latent profile analysis, and network analysis can reveal relationships or interaction characteristics among variables, these approaches generally emphasize estimating independent effects, latent structures, or relational networks among variables [21,22]. They are therefore limited in directly addressing configurational questions, such as which combinations of motor skill conditions are associated with relatively higher levels of executive function. This limitation limits our understanding of the complex combination patterns among gross motor skill domains and makes it difficult for may help identify which combinations of motor skill domains may represent typical patterns associated with higher executive function performance. Synergetic systems theory provides a useful perspective for understanding such complex relationships by suggesting that the overall characteristics of a system may emerge from interactions among its subsystems rather than from the simple accumulation of individual components [23]. Furthermore, the principle of equifinality indicates that similar outcomes may be associated with different combinations of contributing conditions [24]. From this perspective, fuzzy-set qualitative comparative analysis (fsQCA), an approach based on configurational thinking, provides an appropriate methodological framework for exploring complex combinations of antecedent conditions associated with executive function [25]. Therefore, the present study employed fsQCA to explore the configurational patterns associated with executive function among preschool children from the perspective of gross motor skill domains. This approach provides new evidence for addressing the aforementioned research gap. Specifically, this study aimed to answer two core questions: (1) Can combinations of gross motor skill domains form configurational patterns associated with executive function levels in preschool children? (2) How do different combinations of gross motor skill domains constitute distinct configurational pathways associated with executive function levels in preschool children?

## 2. Literature review and model construction

### 2.1. Literature review

GMS refer to movements produced by the large muscles or muscle groups of the body [26]. Research indicates that early acquisition and maintenance of a high level of GMS development in preschool children is associated with subsequent motor skill development [10] and cognitive and social-behavioral development [27]. Conversely, children with low levels of GMS development often face health risks such as physical weakness, overweight, and obesity [28]. Thus, GMS development plays a crucial role in early childhood growth. To better assess GMS development in preschool children, Ulrich (2013) revised the Test of Gross Motor Development to its third edition (TGMD-3), which includes 13 motor skills categorized into locomotor and manipulative skills [29]. Zhao Jinghui developed an evaluation framework for motor skill development, proposing three first-level categories—locomotor, non-locomotor, and object control skills—and further specifying second-level indicators including walking, running, jumping, climbing, rolling, throwing, catching, kicking, striking, balancing, and dodging, along with 25 third-level operational indicators [30]. However, as a measurement tool, the TGMD-3 does not include several of these movements, such as walking, climbing, rolling, balancing, and dodging. Therefore, Zheng et al. (2024), building on Zhao’s classification framework and aligning it with the actual test items of the TGMD-3, grouped the 13 assessed skills into six subsystems: running, jumping, throwing, striking, kicking, and catching [31]. The present study adopted this classification approach, as it retains the fundamental distinction between locomotor and object control skills while ensuring consistency with the TGMD-3 measurement content, thereby demonstrating both theoretical grounding and practical applicability.

Research on EF has been evolving, yet there is no unified consensus on its components. Hill (2004) proposed that EF includes planning, cognitive flexibility, inhibition, fluency, and self-monitoring [32]. Karpinski (2008) categorized EF into inhibitory control, working memory, and planning [33]. Anderson (2012) suggested that EF in preschool children consists of self-regulation, impulse control, working memory, and flexibility [34]. Diamond (2013) emphasized that EF comprises working memory, cognitive flexibility, and inhibitory control [35]. Regarding the structure of EF, there are three main perspectives: (1) EF components form a unified whole under a central control system; (2) EF components are independent of each other; and (3) EF is a unified yet separable whole, where components are interconnected in early development but gradually separate with age [36]. Although there are disagreements on the components of EF, the mainstream view considers EF to consist of working memory, inhibitory control, and cognitive flexibility. This study adopts this mainstream view, treating these three components as a unified whole. Based on the theoretical framework described above, the present study selected EF-Touch tasks representing working memory, inhibitory control, and cognitive flexibility as indicators of executive function. Furthermore, executive function theories emphasize both the unity and diversity of executive function, suggesting that different executive components are relatively distinct while also sharing common underlying control processes. Therefore, the scores of individual tasks were standardized and aggregated to construct a composite executive function index, which was used to represent children’s overall executive function level.

Although GMS and EF are distinct domains in child development, previous research has suggested that they are developmentally interconnected. The performance of GMS involves cognitive processes such as movement planning, attention allocation, and action regulation [37]. Similarly, EF involves cognitive control processes that are relevant to goal-directed behaviors and adaptive responses [38]. Moreover, EF is related to cognitive control processes, such as monitoring, planning, and sequencing, that are also involved in successful motor performance [39]. Piaget’s cognitive development theory posits that GMS and cognitive development are interconnected through the thinking that occurs during physical movement [40], suggesting a potential relationship between motor experiences and cognitive development, including EF [41]. Therefore, the development of gross motor skills may be associated with children’s executive function performance. In recent years, systematic reviews and meta-analytic evidence have further supported the relationship between gross motor skills and cognitive development. A systematic review found that motor skill proficiency was consistently associated with cognitive abilities among children aged 4–16 years, with executive function identified as an important cognitive domain related to motor skills [42]. Further review evidence has indicated that fundamental motor skills in preschool children are associated with higher-order cognitive abilities, including executive function, and that different types of motor skills may involve distinct cognitive demands [43]. Moreover, Jylänki et al. (2022) reported in a systematic review that motor skill and physical activity interventions may be associated with improvements in executive function and other cognitive outcomes among children [44]. However, substantial heterogeneity remains in existing studies regarding intervention content, types of motor activities, and measurement approaches. Studies have shown that GMS is associated with EF performance in preschool children to some extent [45], and improvements in EF have also been reported to be associated with better GMS performance [12]. Developmental studies suggest that GMS and EF show overlapping developmental trajectories during early childhood [46] highlighting their potential relationship during this period [22]. Empirical studies further confirm a significant positive correlation between GMS development and EF in young children [13].

The theory of sensitive periods in childhood emphasizes the crucial role of appropriate environments and educational methods in fostering children’s developmental capacities [47]. During preschool children, both GMS development and EF are considered to be in sensitive periods [45]. Therefore, implementing interventions during this stage aligns with the principles of physical and cognitive development, representing a scientifically sound and holistic approach.

Studies by Brann (2024), Bugos (2017), and Hsiao (2025) have implemented short-term music programs, mindfulness courses, and gesture recognition activities, respectively, and all reported significant improvements in preschool children ‘s EF [48–50]. These findings highlight the potential value of targeted EF interventions during this sensitive period.

Some researchers have simultaneously focused on GMS and EF, conducting relevant interventions among preschool children. Findings indicate that GMS interventions during the sensitive period can significantly enhance both GMS and EF [51,52]. Alesi (2014) and Hudson (2021) consistently found that physical activity interventions incorporating locomotor and manipulative movements led to substantial improvements in EF among preschool children [18,53]. Xiong (2017) conducted structured physical activities involving movements such as kicking and observed significant gains in EF [54]. Similarly, Zhao (2020) implemented functional training programs that included running, jumping, catching, and throwing, resulting in notable EF enhancement [55]. Additionally, Bai (2022) and colleagues organized group play interventions involving jumping, running, and kicking, and found significant improvements across multiple EF domains, including working memory and inhibitory control [56].

In summary, current interventions targeting preschool children ‘s EF primarily include approaches such as music, mindfulness, gesture recognition, as well as GMS interventions involving a variety of movement types—have been reported to improve EF outcomes among preschool children. Among these, studies employing combinations of multiple GMS in intervention design provide valuable insights for the present research, particularly in addressing the first research question: whether subsystems of gross motor skills can form configurations associated with higher EF in preschool children. However, although some research has utilized GMS as an intervention component, few studies have specifically focused on the sensitive period of early childhood to examine the configurational associations between different motor skills and EF performance during early childhood. Therefore, drawing upon the theory of sensitive periods, the present study aims to explore how combinations of gross motor activities are associated with higher EF levels in preschool children. Furthermore, it seeks to analyze possible configurational patterns through which specific GMS combinations are associated with relatively higher EF levels, thereby offering preliminary theoretical insights and practical references for early childhood educators in designing developmentally appropriate interventions.

### 2.2. Model construction

Based on the synergistic systems theory, synergistic effects, and the principle of equifinality outlined in the Introduction, this study constructed a configurational analytical model, as shown in Fig 1.

**Fig 1:**
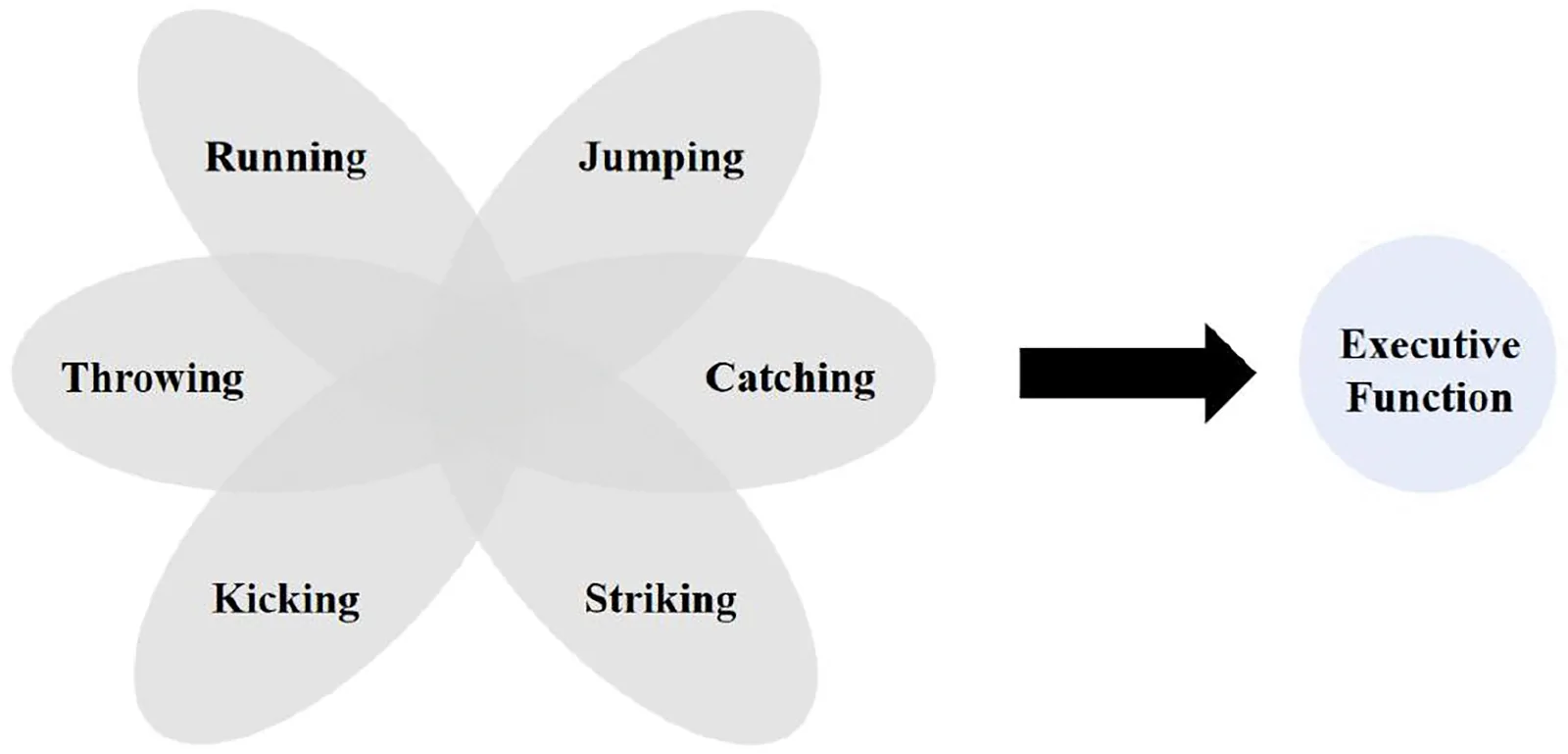
Configurational analytical model of gross motor skill subsystems and executive function. The model presents the six gross motor skill subsystems as antecedent conditions and executive function as the outcome variable for fsQCA analysis.

Systems theory emphasizes that overall system functioning arises from interactions among subsystems rather than the simple aggregation of isolated elements. This perspective guided the present study to decompose gross motor skills into theoretically meaningful subsystems and to incorporate them simultaneously into the analytical framework. The classification of gross motor skill subsystems adopted in this study is grounded in motor development theory, which distinguishes between locomotor and object control skills, as well as in cognitive demand theory, which differentiates movements based on gradients of cognitive load. These subsystems exhibit internal homogeneity and external heterogeneity in terms of biomechanical characteristics and cognitive processing demands, which supports their conceptualization as theoretically meaningful components within the motor skill system.

In line with this perspective, the six subsystems of gross motor skills used in this study conform to the distinction between locomotor and object control skills. Specifically, running and jumping are categorized as locomotor skills, whereas throwing, catching, kicking, and striking are classified as object control skills. Moreover, these movements differ in their cognitive demands, ranging from rhythm control and spatial judgment to hand–eye coordination and real-time prediction. This classification ensures that the six subsystems are theoretically meaningful and analytically distinguishable within the system.

Synergy theory further suggests that the relationships among multiple subsystems may reflect system-level patterns that cannot be fully captured by examining each subsystem independently. Accordingly, this study shifted the analytical focus from the independent relationships of single movements to the configurational relationships of movement combinations. This perspective aligns closely with the configurational logic of fuzzy-set qualitative comparative analysis (fsQCA), which treats combinations of conditions as the unit of analysis and identifies configurations associated with a given outcome.

The principle of equifinality further indicates that multiple different combinations of subsystems may be associated with the same outcome pattern. Therefore, this study employed fsQCA to identify multiple configurational pathways associated with executive function in preschool children.

Based on the theoretical foundations outlined above, this study proposes the following research expectations. Expectation 1: Based on systems theory, higher levels of executive function in preschool children are not expected to be uniquely characterized by a single gross motor skill subsystem, but are more likely to be associated with the combination of multiple motor skill subsystems. Therefore, a single motor skill subsystem is unlikely to constitute a necessary condition for relatively higher executive function membership. Expectation 2: Based on synergistic effects, combinations of locomotor and object control skills may better reflect the overall functioning of the gross motor skill system than a single type of movement. The configurational relationships among different motor skill subsystems may constitute sufficient conditions associated with relatively higher executive function membership. Expectation 3: Based on the principle of equifinality, different combinations of gross motor skill subsystems may form multiple functionally equivalent configurational pathways. In other words, higher levels of executive function in preschool children may not be restricted to a single movement combination, but may instead show configurational characteristics involving multiple coexisting pathways.

In summary, this model treated six gross motor skill subsystems as antecedent conditions and executive function as the outcome variable, and used fsQCA to identify configurational pathways associated with executive function, thereby operationalizing the integration of systems theory and equifinality within the research design.

## 3. Research design

### 3.1. Research methods

In the 1980s, American sociologist Ragin (2009) first proposed the Qualitative Comparative Analysis (QCA) method as a case-oriented approach [57]. QCA is a method that integrates qualitative case analysis with quantitative variable interpretation, examining complex configurational relationships between conditional variables and outcome variables across different cases, thereby addressing analyses that traditional quantitative methods cannot handle [58]. QCA is divided into three forms: Clear-Set Qualitative Comparative Analysis (csQCA), Multi-Value Qualitative Comparative Analysis (mvQCA), and Fuzzy-Set Qualitative Comparative Analysis (fsQCA) [59]. This study adopts the fsQCA method to examine conjunctural relationships and equifinality patterns, which better address the research questions. Compared with traditional statistical methods, fsQCA has greater methodological suitability for the present study. First, Multiple regression primarily focuses on estimating the net effect of individual variables after controlling for other factors. Although it is suitable for examining whether a specific motor skill domain has an independent predictive association with executive function, it is less capable of directly identifying the overall configurational patterns formed by the co-occurrence of multiple motor skill domains. Second, although interaction models can test interactions among variables, including a large number of two-way or higher-order interaction terms would make the model complex, reduce statistical power, and create difficulties in interpretation, particularly because this study includes six motor skill subsystems and has a relatively limited sample size. Third, structural equation modeling is more suitable for testing predefined linear pathways or latent variable structures, Latent profile analysis is primarily used to identify subgroups of individuals with similar characteristics. However, both approaches are limited in directly examining configurational relationships formed by the presence or absence of multiple conditions. In contrast, fsQCA treats combinations of conditions rather than individual variables as the unit of analysis, allowing researchers to examine conjunctural relationships, causal asymmetry, and multiple sufficient configurations (equifinality). Therefore, given that the present study aims to explore how gross motor skill domains combine into different patterns associated with executive function, fsQCA represents a methodologically appropriate approach.

First, Concurrent Causality. This approach avoids examining the association between a single conditional variable and the outcome variable in isolation. Instead, it examines how conditional variables are combined within configurations associated with the outcome variable. By analyzing the necessary conditions and configurations of the antecedent conditions for EF in preschool children, this method explores how GMS subsystems are configured in association with EF, thereby answering Research Question 1.

Second, Equifinality Hypothesis. This hypothesis posits that multiple configurations may be associated with the same outcome pattern, meaning that similar outcome patterns may correspond to different combinations of conditions or produce an unintended outcome. Unlike traditional analytical methods that seek a single optimal solution, fsQCA can reveal multiple configurations of GMS subsystems associated with higher EF levels in preschool children, thereby answering Research Question 2.

### 3.2. Participants

The study was conducted between September and October 2024, with children aged 3–6 years recruited from a kindergarten in Shanghai, China. The primary caregivers of all participating children were informed of the study’s purpose, procedures, and potential risks, and provided written informed consent. This study was approved by the Human Research Ethics Committee of Guangzhou Sport University (Approval No.: 2024LCLL-72). The exclusion criteria were as follows: (1) children with organic diseases or any mental disorders (as reported by parents); and (2) children with intellectual disabilities (as reported by parents).

After the initial data collection, to ensure that the sample adequately represented children with varying developmental levels and to meet the requirement of case heterogeneity in fsQCA, this study followed the principles of “maximum similarity” and “maximum difference” in qualitative comparative analysis for case selection. This approach allowed for the retention of individual case characteristics while identifying shared patterns across cases [25]. Based on these criteria, 67 children were selected from those who completed the initial assessments as the final analytical sample, including 34 boys (50.7%) and 33 girls (49.3%). This sample size is consistent with recommendations for medium-sized samples in fsQCA and allows for the identification of stable configurational patterns while maintaining case diversity.

### 3.3. Variable measurement

#### 3.3.1. GMS development test.

The TGMD-3, developed by Dr. Ulrich and revised in 2013, was used to assess the level of GMS development in preschool children [29]. The TGMD-3 has demonstrated good reliability and validity and has been validated in multiple countries, including the United States, Brazil, and Spain. In China, Diao et al. (2018) conducted a localization study of the scale, reporting an internal consistency reliability of 0.81 and an inter-rater reliability of 0.983. Confirmatory factor analysis indicated that the NNFI was close to 0.95, while both the CFI and GFI exceeded 0.95, suggesting that the TGMD-3 has strong reliability and validity in the Chinese context. Based on these findings, normative data for preschool children’s fundamental movement skills in Shanghai were established [60]. Therefore, this study adopted the TGMD-3 as the measurement tool for assessing fundamental movement skills in preschool children. The TGMD-3 consists of two subtests: the Locomotor Subtest and the Object Control Subtest, comprising a total of 13 specific tasks. Each task has 3−5 criteria, with each criterion scored as “1” if completed and “0” if not completed. Each task is performed twice, and the score for each task is the sum of the two attempts. The total possible score is 100 points. The Locomotor Subtest includes six tasks: running, galloping, hopping, skipping, horizontal jumping, and sliding, with a maximum score of 46 points. The Object Control Subtest includes seven tasks: two-hand striking of a stationary ball, forehand striking of a self-bounced ball, one-hand stationary dribbling, two-hand catching, kicking a stationary ball, overhand throwing, and underhand throwing, with a maximum score of 54 points. Based on the original movement structure of the TGMD-3, this study followed the classification approach used in previous research and grouped the 13 movement tasks into six gross motor skill subsystems: running, jumping, throwing, catching, kicking, and striking. It should be noted that the six subsystems in this study were not newly constructed latent dimensions. Rather, they were operational classifications based on the actual test content of the TGMD-3 and existing movement classification frameworks. Because the different movement tasks in the TGMD-3 have clear motor characteristics, this study emphasizes their functional classification as condition variables in fsQCA, rather than treating them as a reflective scale structure in the traditional psychometric sense. Therefore, this study did not further use factor analysis to validate the structure of the six subsystems. To evaluate the appropriateness of aggregating individual tasks into functional motor skill domains, internal consistency analyses were conducted for domains consisting of multiple tasks. For domains represented by a single task, Cronbach’s α was not calculated because internal consistency estimation requires multiple measurement indicators. During the measurement process, preschool children first watch a demonstration video of each task. They then perform the task three times: the first attempt is a trial, and the latter two are formal measurements. This procedure is repeated for all 13 tasks, with assessors scoring the performances on-site. Verbal encouragement is allowed during the task performance, but no feedback on whether the task criteria were met is provided.

#### 3.3.2. EF Test.

The EF-Touch, a computerized assessment of EF developed by Willoughby in 2013, was used to assess preschool children’s executive function. It has been administered to more than 3,000 children aged 3–6 years, demonstrating a test–retest reliability of up to 0.95. Confirmatory factor analysis has also shown that the CFI exceeds 0.95, indicating that the EF-Touch has good reliability and validity [61]. The software includes multiple measurement tasks, each lasting 3–8 minutes. The tasks are as follows: ①Inhibitory Control Tasks: Arrows, Stroop, Pig. ②Working Memory Tasks: House, Pick the Picture, Farmer. ③Cognitive Flexibility Task: Something the Same. Based on the classical three-component model of EF (inhibitory control, working memory, and cognitive flexibility), and considering the cognitive developmental characteristics of preschool children, this study used the EF-Touch task battery to assess EF. EF-Touch consists of multiple computerized tasks designed to measure different dimensions of EF. In studies of preschool children, researchers typically select a subset of tasks from the battery based on research purposes and participant characteristics to reduce testing time and minimize the effects of fatigue on measurement outcomes [62]. Accordingly, this study selected representative tasks with sound psychometric properties from the EF-Touch battery: the Arrows and Pig tasks were used to assess inhibitory control, the House task was used to assess working memory, and the Something the Same task was used to assess cognitive flexibility. Previous studies have shown that these tasks can reliably reflect the corresponding dimensions of EF in preschool samples and demonstrate good reliability and structural validity [63]. In contrast, some EF-Touch tasks (e.g., Stroop, Pick the Picture, and Farmer) overlap with the selected tasks in terms of the constructs assessed and were therefore not included in this study [64]. To evaluate the reliability of the composite EF measure, internal consistency analysis was conducted using Cronbach’s α. Given that executive function in preschool children is still in a stage of rapid development and gradual differentiation, inhibitory control, working memory, and cognitive flexibility have distinct theoretical meanings but often show relatively strong structural coherence and common developmental characteristics during the preschool years. Garon et al. (2008) noted that executive function in preschool children can be understood as a unified construct with partially separable components [65]. Therefore, when examining the overall level of executive function development in preschool children, previous studies have often used multiple tasks or dimensions to jointly reflect executive function performance [66]. Meanwhile, the primary purpose of the present study was not to compare dimension-specific pathways for different components of executive function, but to examine, from a configurational perspective, the relationship between combinations of gross motor skill subsystems and the overall level of executive function in preschool children. Considering that fsQCA is typically conducted around a single outcome set, treating inhibitory control, working memory, and cognitive flexibility as separate outcome variables would substantially increase model complexity and reduce the clarity of configurational interpretation. Based on these theoretical and methodological considerations, and following previous research practices, this study averaged the standardized scores of inhibitory control, working memory, and cognitive flexibility to construct a composite index of executive function, reflecting the overall executive function performance of preschool children. The assessment of executive function is shown in Fig 2.

**Fig 2:**
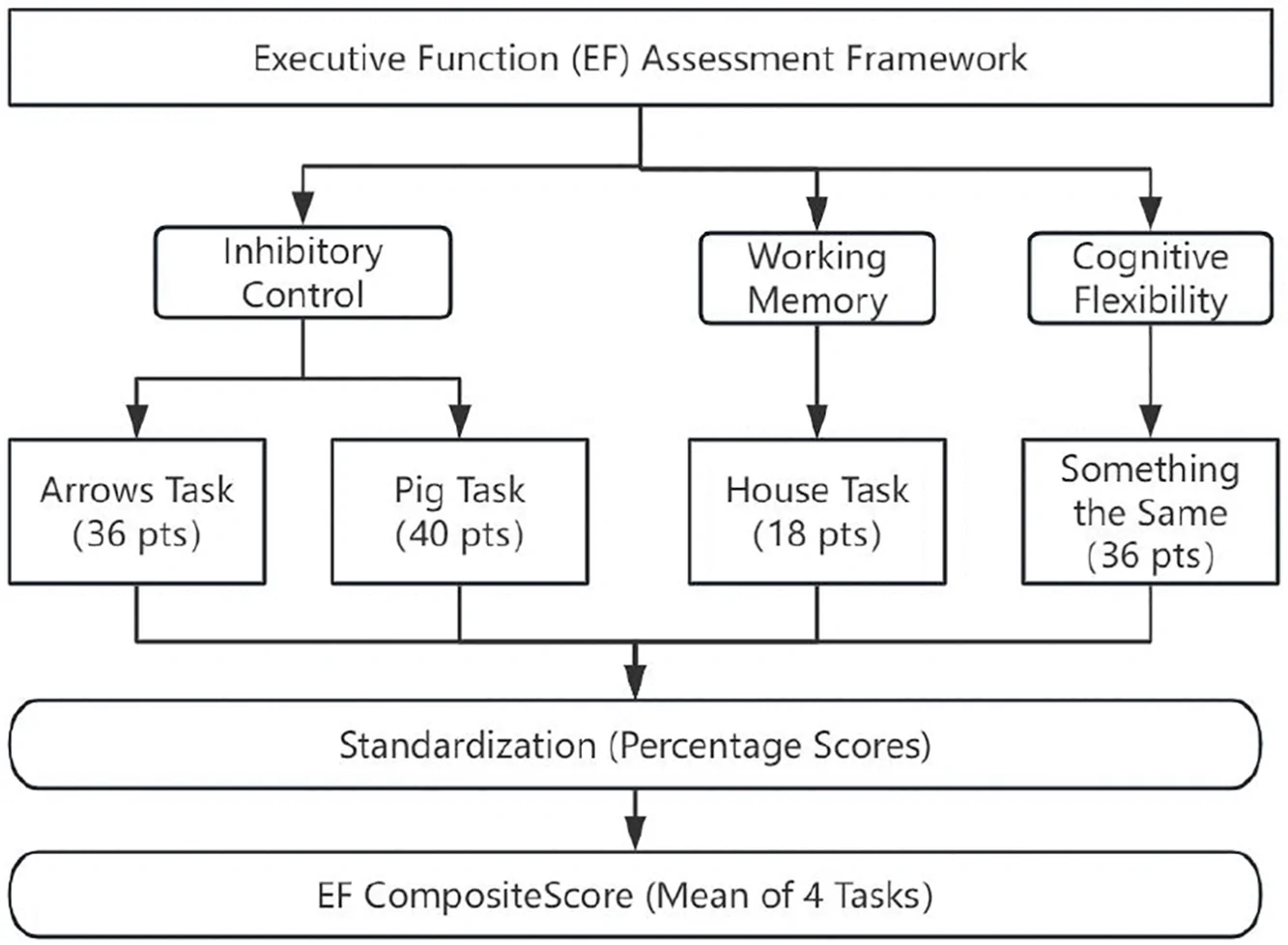
Assessment framework of executive function. The framework shows the three components of executive function (inhibitory control, working memory, and cognitive flexibility) and the corresponding EF-Touch tasks used to construct the composite executive function index.

In the inhibitory control testing, during the Arrows task, participants sit in a chair with their hands placed on the table and are instructed to touch the green button based on the arrow directions displayed on the touch screen. The computer screen provides corresponding instructions (operated by the assessor), while the touch screen shows arrows and buttons (operated by the participant). Participants make judgments based on the touch screen content and touch the green button accordingly. The task includes 8 practice trials (not scored), followed by 36 test trials, with each correct response earning 1 point for a maximum score of 36. In the Pig task, participants sit in a chair with their dominant hand placed on the table and are instructed to skip the green button when a pig image appears and press it otherwise. The computer screen provides corresponding instructions, and the touch screen displays various animal images. First, participants complete an animal identification phase by clicking on the images as instructed by the assessor. Next, participants focus on the touch screen content, make judgments, and decide whether to touch the green button. The task includes 8 practice trials (not scored), followed by 40 test trials, with each correct response earning 1 point for a maximum score of 40.

In the working memory testing, during the House task, participants sit in a chair and are required to remember the animal and color that appear on the touch screen. After the image disappears, they are asked to recall the animal or color that was just shown. The computer screen displays corresponding instructions, while the touch screen shows a house containing an animal and a color. Participants are asked if they remember the animal and color, and after both disappear, they are prompted to recall the animal or color after a brief delay. The task includes 4 practice trials (not scored), followed by 18 test trials, with each correct response earning 1 point for a maximum score of 18. Additionally, after every 3 test trials, a new house is added to the touch screen, progressively increasing the difficulty of the task.

In the cognitive flexibility testing, during the Some The Same task, participants sit in a chair and select images from the touch screen that share consistency in color, shape, or size based on the displayed content. The computer screen provides corresponding instructions, while the touch screen shows images requiring identification of color, shape, and size. Participants click on the appropriate images as instructed by the assessor. Next, the touch screen displays three images, and participants select one that is consistent with the image on the far left. After completing this round, the touch screen again shows three images, and participants must select two images that share consistency. The task includes 5 practice trials (not scored), followed by 30 test trials, with each correct response earning 1 point for a maximum score of 30.

### 3.4. Procedure

The GMS assessment was conducted in either indoor or outdoor settings. Assessors arrived at the testing site 30 minutes in advance to complete equipment setup. Marking lines were arranged to clearly indicate the starting and ending points of each movement. All testing materials, scoring sheets, and writing tools were prepared in advance. Assessors communicated with classroom teachers to confirm the list of participating children, and three children were brought into the testing area at a time.

Before testing began, assessors recorded basic information for each child and provided a brief explanation of the task rules while demonstrating the required movements. For children who had difficulty understanding, one to two additional demonstrations were provided. Each child completed one practice trial followed by two formal trials for each of the 13 movement tasks. Adequate preparation time was provided between tasks to ensure the accuracy of the measurements. During testing, assessors provided verbal encouragement but did not give feedback on performance to maintain objectivity. If a child showed unwillingness to participate or any unexpected situation occurred, the assessment was immediately terminated and the child was returned to the classroom by the teacher. To ensure reliability and consistency, all gross motor assessments were conducted by the same trained evaluator. In addition, to minimize measurement bias, the evaluator remained blinded to other study variables, ensuring independence and objectivity throughout the assessment process.

EF data were collected in a quiet environment. Assessors prepared the testing equipment in advance, including a table, chairs, and a touchscreen device, ensuring that all equipment functioned properly. Assessors first confirmed the list of participating children with the classroom teacher, and one child was brought into the testing area at a time. Upon entry, the assessor recorded the child’s basic information (e.g., class and identification number) and provided a brief introduction to the four tasks (games) to ensure comprehension. If needed, one to two demonstrations were provided. After completing each task, the child proceeded to the next until all tasks were finished. During testing, assessors minimized interaction with the child to avoid influencing performance and continuously monitored the computer-based procedures. The assessor remained blinded throughout the process and did not have access to other variables or background information about the child, ensuring objectivity. If a child did not understand the task rules, additional explanations were provided until comprehension was achieved.

### 3.5. Data analysis

This study primarily employed fsQCA, with data analyzed using SPSS 26.0 and fsQCA 4.1 software. The present study employed the direct calibration method in fsQCA 4.1 to transform raw data into fuzzy-set membership scores. This approach is based on three qualitative anchors: the point of full membership (95%), the crossover point (50%), and the point of full non-membership (5%), through which the original variable values are converted into fuzzy-set membership scores ranging from 0 to 1. The calibration process follows a logistic function:


$$\begin{array}{c} M\;=\frac{\text{1}}{\text{1}+e^{-\frac{x-c}{s}}} \end{array}$$

Where, *M* represents the calibrated fuzzy-set membership score, *x* represents the original value of the variable, *c* represents the crossover point, and *s* represents the slope parameter of the transformation function. Based on the specified qualitative anchors, fsQCA 4.1 automatically estimates the transformation parameters and converts the original data into fuzzy-set membership scores. The analytical procedure consisted of the following steps:

First, data preprocessing was conducted. Due to differences in scoring methods between the TGMD-3 and the EF-Touch assessment, all raw scores were converted into percentage scores to facilitate comparison and calibration. For GMS, the conversion formula was: percentage score = (actual score / total score for each task) × 100. After standardization, the 13 TGMD-3 items were aggregated into six condition variables based on the classification proposed by Zheng et al. (2024). Specifically, the average scores of running, galloping, skipping, and sliding were used to represent running skills; hopping and standing long jump for jumping skills; two-hand striking a stationary ball, forehand striking a dropped ball, and one-hand dribbling for striking skills; two-hand catching for catching skills; stationary kicking for kicking skills; and overhand throw and underhand throw for throwing skills. Because the original TGMD-3 test includes different numbers of tasks across movement categories, the number of tasks included in each motor skill subsystem was not entirely consistent. To reduce the influence of differences in maximum scores and task numbers across movement items, this study first converted each movement task score into a percentage score and then calculated the average score within each subsystem, in order to improve the comparability of scores across different subsystems as much as possible.

For EF, task performance was automatically recorded by the system. As the total scores differed across the three dimensions, standardization was also applied. For example, the standardized score for inhibitory control was calculated as: number of successful trials / total number of trials × 100. After standardization, the scores of inhibitory control, working memory, and cognitive flexibility were averaged to generate a composite EF score, representing the overall level of executive function. Subsequently, descriptive statistics (mean, standard deviation, maximum, and minimum) were calculated for all variables using SPSS.

Second, data calibration was conducted, which is a key step in fsQCA. Calibration transforms raw data into fuzzy-set membership scores ranging from 0 to 1 [67]. This study adopted the direct calibration method and set three qualitative anchors based on recommendations by Fiss [68]. Because there are currently no unified developmental thresholds or widely accepted cut-off criteria for the six TGMD-3 gross motor skill subsystems and the EF-Touch composite executive function index, this study followed common practices in existing fsQCA research and used sample percentiles as calibration anchors. In the absence of external standards, this approach allows relative membership scores to be assigned based on within-sample differences, thereby meeting the fsQCA requirement for calibrating set membership. It should be noted that because the calibration anchors were derived from the distribution of the current sample, the resulting set memberships represent relative positions among participants rather than absolute developmental norms or clinical thresholds. Therefore, the classifications of “high executive function” and “high motor skill performance” should be interpreted as sample-specific relative memberships rather than normative or clinically meaningful cut-offs. To reduce the influence of extreme values and ensure sufficient case variation, the 95th percentile, 50th percentile, and 5th percentile of each variable were set as the thresholds for full membership, crossover point, and full non-membership, respectively. Since a membership score of 0.5 cannot be included in the truth table, values of 0.5 were adjusted to 0.501 to avoid case exclusion [69]. The calibration process was conducted using fsQCA 4.1. In this study, EF was treated as the outcome set (“high EF”), with higher membership scores indicating a greater degree of belonging to the set.

Third, necessity analysis was performed. Prior to configurational analysis, each individual condition (and its negation) was tested to determine whether it constituted a necessary condition for the outcome (high EF). A condition was considered necessary if its consistency exceeded 0.90 and its coverage exceeded 0.50; otherwise, it was not regarded as a necessary condition [70].

Finally, configurational analysis was conducted to identify whether combinations of antecedent conditions constituted sufficient conditions for the outcome. A truth table was first constructed based on calibrated fuzzy-set membership scores, identifying all possible combinations of conditions and calculating the number of cases, raw consistency, and proportional reduction in inconsistency (PRI) for each configuration. Following recommendations for small- to medium-sized samples (Ragin, 2009), the frequency threshold was set to 1, the raw consistency threshold to 0.80 [68], and the PRI consistency threshold to 0.70 to ensure robustness [70]. Configurations with a frequency below 1 were coded as logical remainders and excluded from further analysis. Empirical rows with frequency ≥ 1 were further evaluated: configurations that did not meet both consistency thresholds were coded as “0” (contradictory configurations), while those meeting the thresholds were coded as “1” (configurations associated with high EF). The sufficient configurations were then analyzed using the intermediate solution, with counterfactual analysis applied based on theoretical expectations. Only theoretically plausible counterfactuals were retained in the simplification process, ensuring interpretability and theoretical consistency. Following established conventions [57], the intermediate solution was reported as the primary result, with the parsimonious solution used to distinguish core and peripheral conditions within each configuration.

## 4. Results

### 4.1. Data preprocessing

After data preprocessing, the descriptive statistics of all variables are presented in Table 1.

**Table 1:** Descriptive Statistics of Variables.

Variable Type	Variable Name	Mean	Standard Deviation	Maximum	Minimum
Outcome Variable	EF	76.09	12.47	98.01	27.24
Condition Variables	Running-related	55.52	7.56	70.83	40.63
	Jumping-related	27.05	12.13	62.50	0
	Throwing-related	34.14	12.66	62.50	0
	Catching-related	48.26	17.68	100	16.67
	Kicking-related	27.80	21.30	87.50	0
	Striking-related	40.44	11.64	66.94	18.33

Given that preschool years represent a critical developmental period for executive function and gross motor skills, additional analyses were conducted to examine the associations between demographic characteristics and the main study variables. The results showed that age (in months) was not significantly correlated with EF or any of the six GMS components (all p > .05). Furthermore, sex differences analysis indicated that boys and girls did not differ significantly in EF or most GMS components, except for kicking-related skills. Detailed results are presented in Supplementary Table S1.

To examine whether the aggregation of TGMD-3 tasks into functional motor skill domains was empirically supported, internal consistency analyses were conducted. The results showed that multi-item domains demonstrated satisfactory internal consistency, with Cronbach’s α values of 0.937 for running-related skills, 0.905 for jumping-related skills, 0.914 for throwing-related skills, and 0.880 for striking-related skills. Reliability coefficients were not calculated for catching-related and kicking-related skills because these domains were assessed using single tasks.

To evaluate the reliability of the composite EF measure, internal consistency analysis was conducted. The results showed that the four selected EF tasks demonstrated satisfactory internal consistency (Cronbach’s α = 0.905), supporting the reliability of the composite EF score as an indicator of overall executive functioning.

### 4.2. Data calibration

The calibration anchors for each variable are presented in Table 2.

**Table 2:** Calibration Anchors for Each Variable.

Variable Type	Variable Name	Full Membership (95%)	Crossover Point (50%)	Full Non-membership (5%)
Outcome Variable	EF	90.11	79.06	52.31
Condition Variables	Running-related	69.38	56.25	41.67
	Jumping-related	50.00	25.00	12.50
	Throwing-related	53.75	37.50	12.50
	Catching-related	76.67	50.00	16.67
	Kicking-related	75.00	25.00	0
	Striking-related	59.72	39.44	22.00

### 4.3. Correlation analysis of condition variables

A correlation analysis was conducted among the six subsystems of the condition variables, and the results are presented in Table 3. Previous studies have suggested that multicollinearity may be a concern when correlation coefficients exceed 0.70 [71]. In the present study, all correlation coefficients were well below this threshold, indicating that there was no serious multicollinearity among the variables. In fsQCA, low correlations among condition variables help reduce the risk of spurious configurations caused by substantial overlap between variables, thereby enhancing the validity and interpretability of the identified configurational pathways. Therefore, the six GMS subsystems selected in this study were appropriate for inclusion in the subsequent configurational analysis.

**Table 3:** Pearson correlation matrix of gms variables (N = 67).

Variables	1	2	3	4	5	6
1. Running	1					
2.Jumping	0.075	1				
3.Throwing	−0.044	−0.066	1			
4.Catching	−0.016	0.002	0.028	1		
5.Kicking	−0.043	−0.165	−0.122	−0.096	1	
6.Striking	0.062	−0.043	0.125	−0.041	−0.215	1

### 4.4. fsQCA results

#### 4.4.1. Necessary condition analysis.

Necessary condition analysis aims to determine whether a specific condition variable is an essential prerequisite for triggering the outcome variable. Before conducting configuration analysis, each condition variable must be tested for necessity. Necessity condition analysis provides the consistency and coverage scores for each individual condition. The consistency score indicates the degree to which a condition is a subset of the outcome, while the coverage score reflects the extent to which the condition explains the outcome. The results of the necessary condition analysis for preschool children’s EF are presented in Table 4. No condition variable met the criteria for consistency level and coverage required to be classified as a necessary condition. This indicates that no single motor skill subsystem constitutes a necessary condition for relatively higher EF membership, thereby supporting Research Expectation 1. Therefore, it is essential to include all motor skill elements for further configuration analysis to explore how their combinations are associated with preschool children’s EF.

**Table 4:** Results of Necessary Condition Analysis for Preschool Children’s EF.

Condition Variable	EF	
	Consistency	Coverage
Running-related	0.655	0.716
~Running-related	0.654	0.663
Jumping-related	0.638	0.688
~Jumping-related	0.629	0.645
Throwing-related	0.676	0.765
~Throwing-related	0.616	0.606
Catching-related	0.613	0.669
~Catching-related	0.675	0.685
Kicking-related	0.594	0.665
~Kicking-related	0.679	0.674
Striking-related	0.728	0.757
~Striking-related	0.560	0.597

Note: The symbol “~” represents the logical NOT operation.

#### 4.4.2. Path analysis.

Based on the calibrated fuzzy-set data, a truth table was constructed, yielding a total of ${\text{2}}^{\text{6}}=\text{6}\text{4}\;$logically possible combinations of conditions. The truth table was coded according to the rules described in Section 3.5. After removing logical remainders (50 rows with zero cases), 14 configurations with empirical cases remained. According to the coding criteria, six configurations met both the raw consistency threshold (≥ 0.8) and the PRI threshold (≥ 0.7) and were coded as “1” (high EF), while the remaining eight configurations that did not meet these thresholds were coded as “0.” Due to space limitations, presents only the sufficient configurations and contradictory rows; detailed results are shown in Table 5.

**Table 5:** Truth Table for Sufficient Configurations and Contradictory Rows.

Num	PLfs	TLfs	PDfs	PJfs	Tfs	TZfs	Cases	Raw Consistency	PRI Consistency	Result
1	1	1	1	1	0	1	1	0.9498	0.8414	1
2	1	1	1	0	0	1	1	0.9343	0.7813	1
3	1	0	0	1	1	1	1	0.9547	0.7791	1
4	0	1	1	1	1	1	1	0.9209	0.7480	1
5	1	0	1	0	1	0	1	0.9342	0.7020	1
6	0	1	1	1	0	1	1	0.9034	0.7017	1
7	1	1	1	0	0	0	2	0.8972	0.6975	0
8	0	0	1	0	1	0	1	0.8996	0.6850	0
9	1	0	0	0	1	1	1	0.9256	0.6115	0
10	1	0	0	0	0	1	2	0.8624	0.5548	0
11	0	1	0	0	1	0	3	0.7505	0.3457	0
12	1	1	0	0	1	0	1	0.7958	0.3141	0
13	0	0	0	1	1	0	2	0.8020	0.2604	0
14	1	0	0	0	1	0	1	0.8082	0.2566	0

The sufficient configurations identified above were further analyzed to derive configurational pathways, as presented in Table 6. In terms of consistency and coverage, following the criteria proposed by Ragin and Fiss [57], a solution is considered acceptable if the consistency exceeds 0.75 and the coverage exceeds 0.20. In this study, all three identified pathways met and exceeded these thresholds, indicating that the configurational results are acceptable consistency and coverage in explaining the observed outcome patterns. These findings are consistent with Research Expectations 2 and 3.

**Table 6:** Configuration Paths Associated with Higher EF.

Condition Variables	EF		
	A	B	C
Running-related	●	★	★
Jumping-related	★	□	□
Throwing-related	★	□	★
Catching-related	--	□	★
Kicking-related	□	★	●
Striking-related	★	★	□
Coverage	0.287	0.238	0.227
Unique Coverage	0.084	0.068	0.053
Cases	19	16	15
Consistency	0.938	0.934	0.955
Overall Coverage	0.496		
Overall Consistency	0.906		

To facilitate a clearer interpretation of the role of each condition within the configurational pathways, the symbolic notation follows established conventions in prior research [72]: ★ indicates the presence of a core condition, ● indicates the presence of a peripheral condition, □ indicates the absence of a core condition, and “--” indicates that the condition is irrelevant (i.e., may be either present or absent). Core conditions appear in both the intermediate and parsimonious solutions, whereas peripheral conditions appear only in the intermediate solution.

### 4.5. Robustness tests

To ensure the reliability of the configurational results, robustness tests were conducted from three aspects, following established practices in prior research [73]. First, the frequency threshold was increased from 1 to 2, and the three resulting configurations remained consistent with those obtained in the original analysis. Second, the raw consistency threshold was raised from 0.80 to 0.85, and the resulting configurations still included the original solutions. Third, the calibration anchors were adjusted by setting the thresholds for full non-membership and full membership at the 15th and 85th percentiles, respectively, while keeping the crossover point unchanged; the resulting configurations remained consistent with the original findings. The robustness test that adjusted the calibration anchors was mainly used to examine whether the configurational results were overly dependent on the original percentile-based calibration. The results showed that the configurations generated after adjusting the calibration anchors were largely consistent with the original results, indicating that the configurational pathways identified in this study were not solely dependent on a single calibration scheme. Overall, these results suggest that the identified configurational findings show acceptable robustness under alternative analytical specifications.

## 5. Discussion

Using fsQCA, necessity and configurational analyses identified three pathways associated with higher levels of EF. The overall solution showed a consistency of 0.906 and a coverage of 0.496, indicating that these configurations accounted for approximately 49.6% of the cases. Among the 67 preschool children in this study, approximately 19 cases corresponded to Pathway A, 16 to Pathway B, and 15 to Pathway C. The results indicate that higher levels of executive function in preschool children do not depend on a single gross motor skill subsystem, but are more likely to be associated with combinations of multiple motor skill subsystems. This is consistent with the research expectations proposed earlier based on systems theory, synergistic effects, and the principle of equifinality. Based on the core conditions of each configuration, the three pathways were labeled as follows: (A) Jumping–Throwing–Striking Promotion Type, (B) Running–Kicking–Striking Promotion Type, and (C) Running–Throwing–Catching Promotion Type.

### 5.1. Jumping–Throwing–Striking configuration

Configuration A indicates that the coexistence of higher membership in jumping, throwing, and striking conditions, together with running as a peripheral condition, was associated with relatively higher EF membership. In this configuration, the catching condition is absent, suggesting that jumping, throwing, and striking represented core conditions within this configurational pattern associated with relatively higher EF membership, while catching is less relevant. This pathway accounted for approximately 19 cases, among which about 6 cases were uniquely explained by this configuration. This indicates that, in the present study, 19 children with higher levels of jumping, throwing, and striking skills were associated with higher levels of EF. Additionally, approximately 6 children exhibited higher levels of EF even when other motor skill subsystems were relatively weak.

From the perspective of motor characteristics, this configuration includes multiple types of motor skills with distinct task demands. Jumping-related skills typically require children to maintain postural control, regulate movement rhythm, and organize sequential motor actions [74,75]. Throwing-related skills involve target orientation, force control, and hand–eye coordination [76]. Striking-related skills require children to maintain movement rhythm continuously and adjust their motor performance according to task requirements. Therefore, compared with individual motor skills, this combination may involve more diverse demands for motor control and task regulation. During the completion of these motor tasks, children may need to continuously engage in action planning, attentional monitoring, and behavioral adjustment, which are processes that share certain similarities with the cognitive regulation processes underlying executive function.

### 5.2. Running–Kicking–Striking configuration

Configuration B demonstrates that excellent performance in running, kicking, and striking as core conditions was associated with relatively higher EF membership. In this configuration, strong abilities in running, kicking, and striking was observed even when other motor skill subsystems showed relatively lower membership even when jumping, throwing, and catching development is weaker. The coverage and unique coverage of this configuration were 0.238 and 0.068, respectively. This pathway accounted for approximately 16 cases, among which about 5 cases were uniquely explained by this configuration. This indicates that, in the present study, 16 children with higher levels of running, kicking, and striking skills were associated with higher levels of EF. Additionally, approximately 5 children exhibited higher levels of EF even when other motor skill subsystems were relatively weak.

From the perspective of motor characteristics, this configuration includes both locomotor skills and object-control skills, which may reflect complementary relationships among different motor task demands. Running-related skills primarily involve body movement, rhythm maintenance, and the control of continuous movement, requiring children to continuously adjust their body position and movement speed according to the surrounding environment. Kicking-related skills require children to identify target objects and adjust movement direction, force, and timing based on target location [77]. Striking-related skills require children to maintain movement rhythm and make immediate adjustments according to movement feedback [78]. Therefore, the combination of these motor domains may involve diverse aspects of motor demands, including body control, goal-directed behaviors, and movement feedback regulation. Compared with single-type motor tasks, activities involving multiple motor demands may provide children with richer contexts for motor regulation, requiring them to continuously attend to task goals, adjust movement strategies, and maintain behavioral control during task completion [79]. Therefore, the running-related, kicking-related, and striking-related configuration identified in the present study may represent a characteristic motor skill pattern that co-occurs with relatively higher executive function levels.

### 5.3. Running–Throwing–Catching configuration

Configuration C demonstrates that excellent performance in running, throwing, and catching as core conditions, supplemented by good performance in kicking as a peripheral condition, was associated with relatively higher EF membership. In this configuration, strong abilities in running, throwing, and catching, combined with good kicking skills, was observed in children with relatively weaker performance in other motor skill domains even when jumping and striking development is weaker. The coverage and unique coverage of this configuration were 0.227 and 0.053, respectively. This pathway accounted for approximately 15 cases, among which about 4 cases were uniquely explained by this configuration. This indicates that, in the present study, 15 children with higher levels of running, throwing, and catching skills were associated with higher levels of EF. Additionally, approximately 4 children exhibited higher levels of EF even when other motor skill subsystems were relatively weak.

From the perspective of motor task characteristics, this configuration primarily consists of locomotor skills and object-control skills, reflecting the combination of different types of motor task demands. Running-related skills emphasize rhythm control, spatial positioning, and adjustment of continuous movement during body locomotion; throwing-related skills require children to adjust movement direction, force, and timing according to target location; and catching-related skills require children to continuously monitor changes in the position of moving objects and adjust their motor responses based on external feedback. Therefore, this combination may involve multiple motor task demands, ranging from body movement control and goal-directed actions to adaptation to dynamic environments. Previous research has suggested that motor tasks involving target identification, movement adjustment, and feedback control may share certain characteristics with children’s cognitive regulation abilities [80]. Based on this perspective, the running-related, throwing-related, and catching-related configuration identified in the present study may represent a motor skill combination pattern characterized by relatively higher complexity in motor regulation. Specifically, running-related skills involve continuous body control, throwing-related skills emphasize goal-directed motor organization, and catching-related skills require children to continuously adjust behavioral responses based on dynamic information. The differences in task demands among these three motor domains may allow children to demonstrate more comprehensive motor control and behavioral regulation during motor performance, which may co-occur with relatively higher executive function levels.

### 5.4 Integrated comparative analysis

A comparison of the three configurational pathways reveals that, although they differ in specific movement combinations, all pathways involve the joint participation of locomotor and object control skills. This suggests that EF is not associated with a single type of movement but is more likely linked to the combined presence of different movement types. From a functional perspective, locomotor skills primarily provide dynamic regulation and sustained execution support, whereas object control skills emphasize interaction with external objects, spatial judgment, and hand–eye coordination. These two types of movements differ in their cognitive demands, and when combined, they may form a complementary relationship between “self-movement control” and “external information processing,” thereby being associated with higher EF performance.

In addition, the three pathways exhibit different configurational patterns, indicating that when certain motor subsystems are absent, other combinations can achieve similar outcomes through different functional patterns. For example, these pathways may reflect different combinations of motor task characteristics, although the underlying processes cannot be determined from the present cross-sectional design. This finding further enriches the concept of equifinality, suggesting that different movement combinations may not only vary structurally but also differ in their possible functional characteristics.

Considering the variables selected and the research context, the findings have preliminary practical and developmental implications. In this study, GMS were categorized into six subsystems—running, jumping, throwing, catching, kicking, and striking—representing different functional dimensions of locomotor and object control skills. The configurational results indicate that combinations of these subsystems showed meaningful configurational associations beyond individual movement domains, providing preliminary empirical support for the functional differentiation and combinational analysis of GMS. From a practical perspective, although this study did not implement specific intervention programs, the identified movement combinations offer initial insights for designing physical activities for preschool children. In practice, educators may consider integrating locomotor and object control skills to design diverse and combined activities—for example, incorporating running, throwing, and catching into game-based contexts or developing activities that require both rhythm and coordination—but the effectiveness of such activity designs for EF development requires further experimental verification.

From a developmental perspective, the participants in this study were children aged 3–6 years, a period characterized as a sensitive stage for both EF and GMS development. Given that cognitive structures in early childhood are not yet fully differentiated, the observed configurational patterns may reflect the coexistence of multiple motor skill combinations associated with EF performance during this period. Therefore, the multiple configurational pathways identified in this study may reflect this developmental characteristic, indicating that children may achieve higher levels of EF through different combinations of movements rather than relying on a single developmental pathway.

Although three configurational pathways associated with higher levels of EF were identified, approximately 34 cases were not covered by these pathways. This phenomenon is common in fsQCA studies, as configurational analysis aims to identify typical combinations of conditions associated with an outcome rather than to explain all cases [68]. The unexplained cases may be associated with other combinations of conditions or influenced by factors not included in this study, such as family environment, individual developmental differences, or other educational interventions. Although the functional classification of TGMD-3 tasks provided a useful framework for examining configurational relationships among motor skill domains, the number of tasks contributing to each domain was not equivalent. In particular, catching-related and kicking-related skills were represented by single tasks, which may result in differences in measurement precision across domains. Future studies with larger samples and more comprehensive motor assessments are encouraged to further validate these functional classifications. Therefore, the three pathways identified in this study primarily represent stable and representative patterns of association between GMS combinations and EF within the present sample, preliminary evidence for future research and activity design rather than direct evidence for specific intervention strategies.

## 6. Conclusion and recommendations

By applying fsQCA, with six gross motor skill domains (running-related, jumping-related, throwing-related, catching-related, kicking-related, and striking-related skills) as condition variables, this study explored the configurational patterns associated with executive function performance in preschool children from a configurational perspective. The main findings are as follows.

First, executive function performance was not simply associated with any single gross motor skill domain but demonstrated a configurational pattern involving multiple conditions. Different combinations of motor skill domains may be associated with relatively higher levels of executive function performance, suggesting complex patterns of association between gross motor skills and executive function.

Second, this study identified three configurational pathways associated with relatively higher executive function levels: the “jumping–throwing–striking configuration,” the “running–kicking–striking configuration,” and the “running–throwing–catching configuration.” These configurations accounted for different subsets of cases with relatively high executive function membership, reflecting the principle of equifinality in the relationship between gross motor skill combinations and executive function performance. It should be noted that these configurational pathways do not represent comprehensive explanatory models of executive function development; rather, they characterize typical patterns in which specific combinations of motor skill domains co-occur with executive function performance among some preschool children.

Based on these findings, the design of motor activities for preschool children may consider combinations of different motor skill domains rather than focusing solely on individual motor skills. For example, activities incorporating jumping, throwing, and striking skills, or running, kicking, and striking skills, may provide diverse motor experiences. However, because the present study cannot establish causal relationships, future longitudinal and intervention studies are needed to further examine the relationships between different motor skill combinations and changes in executive function.

## 7. Limitations and future directions

Based on systems theory, synergistic effects, and the principle of equifinality, this study used fsQCA to explore configurational patterns linking gross motor skill subsystems with higher levels of executive function in preschool children, providing preliminary configurational evidence for understanding the relationship between gross motor skills and executive function in early childhood. However, due to limitations in the research design and research conditions, this study has several limitations. First, this study included only 67 children from one kindergarten in a single region. Although the sample size is within the range commonly reported in small- and medium-sized fsQCA studies, the relatively concentrated sample source and limited number of cases may restrict the generalizability and stability of the identified configurations. Therefore, the generalizability of the findings needs to be further validated in more regions, more kindergartens, and larger samples. Second, this study did not conduct replication testing or external validation using an independent sample. Therefore, whether the identified configurational pathways can be stably applied to children from different developmental backgrounds remains to be further examined in future research. Third, this study integrated inhibitory control, working memory, and cognitive flexibility into a composite index of executive function. Although this approach helps examine the relationship between preschool children’s overall executive function and combinations of gross motor skill subsystems, it may also obscure differential associations between specific executive function dimensions and particular motor skill subsystems. Future studies may use inhibitory control, working memory, and cognitive flexibility as separate outcome variables to further conduct dimension-specific configurational analyses. Finally, this study used fsQCA to identify configurational pathways associated with higher levels of executive function based on combinations of condition variables. Although this method helps reveal the combinational relationships among different motor skill subsystems, the results primarily reflect configurational associations among variables and cannot directly demonstrate the effectiveness of these pathways in actual intervention contexts. Therefore, future research may combine longitudinal tracking, experimental intervention, or multiple-case validation to further examine the applicability and stability of these configurational pathways in real educational settings.

## Supporting information

S1 Table Associations of age and sex with executive function and gross motor skill components. (DOCX)
