# Supplementary material for: The configurational pathways associated with executive function in preschool children: An fsQCA analysis based on gross motor skill development
Source: PLoS One. 2026 Oct 26;21(10):e0361476. doi: 10.1371/journal.pone.0361476 (PMC13704824; doi:10.1371/journal.pone.0361476)
Supplement: Supplementary file 1 [file pone.0361476.s001.docx]

Associations of Age and Sex With Executive Function and Gross Motor Skill Components

| Variables | Age (r) | p | Sex (t) | p |
| --- | --- | --- | --- | --- |
| Executive function (EF) | -0.090 | .467 | -0.609 | .545 |
| Running | 0.078 | .529 | -0.810 | .421 |
| Jumping | -0.109 | .381 | 0.858 | .394 |
| Throwing | 0.113 | .361 | -0.625 | .534 |
| Catching | 0.079 | .525 | -1.022 | .311 |
| Kicking | -0.052 | .673 | -2.145 | .036 |
| Striking | -0.103 | .407 | -0.691 | .492 |
